# New insights into *Trypanosoma cruzi* evolution, genotyping and molecular diagnostics from satellite DNA sequence analysis

**DOI:** 10.1371/journal.pntd.0006139

**Published:** 2017-12-18

**Authors:** Juan C. Ramírez, Carolina Torres, María de los A. Curto, Alejandro G. Schijman

**Affiliations:** 1 Laboratorio de Biología Molecular de la Enfermedad de Chagas, Instituto de Investigaciones en Ingeniería Genética y Biología Molecular "Dr. Héctor N. Torres" (INGEBI), CONICET, Buenos Aires, Argentina; 2 Departamento de Microbiología, Inmunología y Biotecnología, Facultad de Farmacia y Bioquímica, Universidad de Buenos Aires, Buenos Aires, Argentina; 3 CONICET, Buenos Aires, Argentina; Instituto de Ciências Biológicas, Universidade Federal de Minas Gerais, BRAZIL

## Abstract

*Trypanosoma cruzi* has been subdivided into seven Discrete Typing Units (DTUs), TcI-TcVI and Tcbat. Two major evolutionary models have been proposed to explain the origin of hybrid lineages, but while it is widely accepted that TcV and TcVI are the result of genetic exchange between TcII and TcIII strains, the origin of TcIII and TcIV is still a matter of debate. *T*. *cruzi* satellite DNA (SatDNA), comprised of 195 bp units organized in tandem repeats, from both TcV and TcVI stocks were found to have SatDNA copies type TcI and TcII; whereas contradictory results were observed for TcIII stocks and no TcIV sequence has been analyzed yet. Herein, we have gone deeper into this matter analyzing 335 distinct SatDNA sequences from 19 *T*. *cruzi* stocks representative of DTUs TcI-TcVI for phylogenetic inference. Bayesian phylogenetic tree showed that all sequences were grouped in three major clusters, which corresponded to sequences from DTUs TcI/III, TcII and TcIV; whereas TcV and TcVI stocks had two sets of sequences distributed into TcI/III and TcII clusters. As expected, the lowest genetic distances were found between TcI and TcIII, and between TcV and TcVI sequences; whereas the highest ones were observed between TcII and TcI/III, and among TcIV sequences and those from the remaining DTUs. In addition, signature patterns associated to specific *T*. *cruzi* lineages were identified and new primers that improved SatDNA-based qPCR sensitivity were designed. Our findings support the theory that TcIII is not the result of a hybridization event between TcI and TcII, and that TcIV had an independent origin from the other DTUs, contributing to clarifying the evolutionary history of *T*. *cruzi* lineages. Moreover, this work opens the possibility of typing samples from Chagas disease patients with low parasitic loads and improving molecular diagnostic methods of *T*. *cruzi* infection based on SatDNA sequence amplification.

## Author summary

*Trypanosoma cruzi*, the causative agent of Chagas disease, is a genetically complex protozoan parasite. *T*. *cruzi* strains have been classified into seven Discrete Typing Units (DTUs), TcI-TcVI and Tcbat, which have been associated with different geographic distribution and transmission cycles. Two major evolutionary models have been proposed to explain the origin of hybrid lineages, but the phylogenetic relationships between the different *T*. *cruzi* DTUs are not completely understood. On the other hand, the molecular markers usually used for DTU identification are found in a low copy number and do not allow the direct typing of biological samples with low parasite burden. However, this goal can be reached using molecular targets with highly repetitive sequences like *T*. *cruzi* satellite DNA (SatDNA), widely used for molecular diagnostics of Chagas disease. This study has been focused on the phylogenetic analysis of SatDNA sequences from DTUs TcI-TcVI aimed to clarifying the origin of *T*. *cruzi* lineages. In addition, our findings open the possibility of typing clinical samples with low parasitic loads and improving SatDNA real-time PCR sensitivity for molecular diagnostics of *T*. *cruzi* infection.

## Introduction

*Trypanosoma cruzi*, the causative agent of Chagas disease, has been subdivided into seven Discrete Typing Units (DTUs), TcI-TcVI and Tcbat, which have been associated with different geographic distribution and transmission cycles [1–4]. Historically, the genetic diversity displayed by *T*. *cruzi* was attributed to predominant clonal evolution [5–7]. However, an increasing number of evidences indicates that natural genetic exchange may be frequent and has had a fundamental role in the evolution of *T*. *cruzi* DTUs [8–11]. Thereby, while the theory of clonal evolution may explain the usual mode of *T*. *cruzi* population expansion, it is widely accepted that hybridization events had an important impact on the current population structure of this parasite, including the existence of hybrid lineages [9,11–14].

Two major evolutionary models have been proposed to explain the origin of hybrid lineages, the "Two Hybridization" [12] and the "Three Ancestor" [13] models. After analyzing nine nuclear loci from 26 isolates representative of *T*. *cruzi* DTUs TcI-TcVI, Westenberger et al. (2005) [12] postulated the hypothesis that an ancient fusion between TcI and TcII strains led, through a loss of TcI/II hybrid heterozygosity and independent clonal evolution, to the origin of TcIII and TcIV; later on a more recent hybridization event between TcII and TcIII strains generated TcV and TcVI by independent clonal evolution. On the other hand, following the analysis of five microsatellite loci and three mitochondrial genes from 75 TcI-TcVI stocks, Freitas et al. (2006) [13] proposed the existence of at least three ancestral lineages (TcI-TcIII) and that two recent and independent genetic exchange events between TcII and TcIII strains resulted in TcV and TcVI; whereas the origin of TcIV could not be fully addressed due to the few isolates analyzed from this DTU.

Recently, a third and more complex scenario has been postulated by Tomasini and Diosque (2015) [14]. Thirteen housekeeping genes from 25 isolates representing *T*. *cruzi* DTUs TcI-TcVI were analyzed, as well as data published by other authors, including those from Westenberger et al. (2005) [12] and Freitas et al. (2006) [13]. They proposed that a common *T*. *cruzi* ancestor diverged into two groups: TcII and TcI-TcIII-TcIV, followed by TcIV separation and diversification into South (TcIV_S_) and North (TcIV_N_) American populations. Subsequently, TcI and TcIII diverged from the previous ancestor and TcIII received TcIV_S_ mitochondrial DNA by multiple introgression events; phenomenon also described by others [9,15]. Finally, as it was proposed for the Three Ancestor model, two recent and independent hybridization events between TcII and TcIII led to the origin of TcV and TcVI [14].

*T*. *cruzi* satellite DNA (SatDNA), widely used as target for molecular diagnostics of Chagas disease [16–20], comprises 195 bp units organized in tandem repeats of about 30 ± 10 kb in some chromosomes [21] and constitutes approximately 5% of parasite genome [22]. Phylogenetic inference from 100 SatDNA sequences from TcI-TcIII and TcVI stocks showed that TcIII and TcVI sequences were distributed into TcI and TcII clusters, supporting the Two Hybridization model [23]. In a more recent network genealogy analysis of 139 SatDNA sequences from TcI-TcIII and TcV-TcVI stocks it was found that all TcIII sequences, including those from Elias et al. (2005) [23], were grouped together with TcI sequences [24]. However, in the light of the Two Hybridization model the authors suggested that TcII SatDNA fingerprints were present in the ancestral TcIII but have been smudged in current TcIII strains, as proposed for other TcII genes [8,12,25].

Herein, we have gone deeper into this matter analyzing SatDNA sequences from *T*. *cruzi* DTUs TcI-TcVI, including TcIV_S_ and TcIV_N_ isolates, for phylogenetic inference. In addition, we performed a signature pattern analysis to identify polymorphic sites associated to specific *T*. *cruzi* lineages and designed new primers for molecular diagnostic purposes.

## Methods

### Parasite isolates and DNA extraction

The *T*. *cruzi* stocks used in this work (Table 1) came from already-existing collections (see Acknowledgments section for details). The DTU classification of these isolates has been previously reported [1,26] and was confirmed using a Multiplex qPCR assay with TaqMan probes targeted to nuclear and mitochondrial genomic markers, as previously described [26]. Epimastigote forms of *T*. *cruzi* stocks were cultured in liver infusion-tryptose medium with 10% fetal calf serum (NATOCOR, Cordoba, Argentina) at 28°C, as previously described [27]. Parasite genomic DNA was purified using the High Pure PCR Template Preparation Kit (Roche Diagnostics, Indianapolis, IN) according to manufacturer instructions for cultured cells.

**Table 1:** Description of *T*. *cruzi* stocks and satellite DNA sequences analyzed in this work.

*T*. *cruzi* stock	DTU	Origin	Host/Vector	No. of sequences	Reference^a^
**Sylvio X10 cl1**	**TcI**	Brazil	*Homo sapiens*	23*	Elias et al., 2005
**K-98**		Argentina	*Homo sapiens*	19	This work
**Col4R**		Colombia	*Homo sapiens*	17	This work
**Dm28c**		Venezuela	*Didelphis marsupialis*	12	This work
**Y**	**TcII**	Brazil	*Homo sapiens*	20*	Elias et al., 2005
**JG**		Brazil	*Homo sapiens*	23	This work
**3869**	**TcIII**	Brazil	*Homo sapiens*	14*	Ienne et al., 2010
**M6241 cl6**		Brazil	*Homo sapiens*	20*	Elias et al., 2005; Ienne et al., 2010
**LL051-P24-Ro**		Argentina	*Canis familiaris*	18	This work
**4167**	**TcIV**	Brazil	*Rhodnius brethesi*	19	This work
**Am64**		Brazil	*Homo sapiens*	19	This work
**Dog Theis**		USA	*Canis familiaris*	13	This work
**115**	**TcV**	Brazil	*Homo sapiens*	10*	Ienne et al., 2010
**B147**		Brazil	*Homo sapiens*	13*	Ienne et al., 2010
**NR cl3**		Chile	*Homo sapiens*	14*	Ienne et al., 2010
**CL Brener**	**TcVI**	Brazil	*Triatoma infestans*	20*	Elias et al., 2005
**RA**		Argentina	*Homo sapiens*	20	This work
**VD**		Argentina	*Homo sapiens*	24	This work
**Tulahuen**		Chile	*Homo sapiens*	17	This work

DTU: Discrete Typing Unit

^a^Articles from where SatDNA sequences were obtained

*Sequences downloaded from GenBank

### Cloning and sequencing of 195 bp satellite DNA

Ten μg of genomic DNA was digested with FastDigest SacI restriction enzyme (Thermo Scientific, Waltham, MA). The 195 bp fragments were purified from agarose gels using Wizard SV Gel and PCR Clean-Up System (Promega, Madison, WI) and cloned in the pBluescript SK(-) plasmid (Stratagene, La Jolla, CA) at the SacI site. After SatDNA qPCR confirmation [20], the positive clones were sequenced using M13 forward primer (MACROGEN, Seoul, Korea). In addition to the 201 sequences obtained in this work, 134 SatDNA sequences from eight *T*. *cruzi* stocks were downloaded from GenBank (Table 1).

### Phylogenetic analysis

Sequences were aligned using ClustalX v2.1 [28] and edited with BioEdit v7.0 [29]. Phylogenetic tree was built using Bayesian inference with MrBayes v3.2 [30]. Analysis was performed using an appropriate substitution model according to the Akaike Information Criterion, estimated with jModelTest v2.1 [31]. Analysis was run for 100 million generations and sampled every 50000 generations, in the CIPRES Science Gateway server [32]. Convergence was assessed by effective sample size values higher than 200 using Tracer v1.6 [33], and the initial 10% sampling was discarded as burn-in. In addition, genetic distances within and between phylogenetic clusters and *T*. *cruzi* DTUs sequences were estimated using an appropriate substitution model according to the Akaike Information Criterion with MEGA v7.0 [34]; standard error was estimated by bootstrap analysis (1000 replicates).

### Signature pattern analysis

Sequences were analyzed using VESPA v1.0 [35] to identify SatDNA type signature patterns associated to phylogenetic clusters. To validate our findings, the consensus sequence of each *T*. *cruzi* stock was obtained with BioEdit v7.0 [29], considering all nucleotides present in at least 20% sequences for each polymorphic site, and classified into SatDNA types according to its similarity to a particular signature pattern. Finally, the SatDNA type of each consensus sequence was compared with the corresponding DTU of each *T*. *cruzi* stock.

### Primers design and SatDNA qPCR analysis

The graphic representation of the SatDNA consensus sequence of all *T*. *cruzi* stocks was obtained using WebLogo [36]. Based on it, specific SatDNA primers cruzi1c (5'-TGAATGGYGGGAGTCAGAG-3') and cruzi2c (5'-ATTCCTCCAAGMAGCGGAT-3') were designed for being used in a real-time PCR assay together with cruzi3 TaqMan probe [17]. Primer properties and specificity were verified using OligoAnalyzer v3.1 (available at http://www.idtdna.com) and BLAST search against non-redundant database [37], respectively.

The novel cruzi1c/cruzi3/cruzi2c SatDNA qPCR assay was compared with the validated SatDNA qPCR method [20]. Both qPCR assays were performed as previously described [20], in simplex format, and challenged against genomic DNA from 12 *T*. *cruzi* stocks [TcI (Sylvio X10 cl1 and K-98 stocks), TcII (Y and JG stocks), TcIII (M5631 cl5 and LL051-P24-Ro stocks), TcIV (4167 and Am64 stocks), TcV (MN cl2 and PAH179 stocks), and TcVI (CL Brener and RA stocks)], in concentrations that ranged from 1.0 to 0.0625 fg/μL. Both amplifications were carried out simultaneously in an ABI7500 real-time PCR device (Applied Biosystems, Foster City, CA).

## Results

Two hundred and one distinct SatDNA copies from three TcI (K-98, Col4R, and Dm28c), one TcII (JG), one TcIII (LL051-P24-Ro), three TcIV (4167, Am64, and Dog Theis), and three TcVI (RA, VD, and Tulahuen) stocks were cloned and sequenced (Table 1). These sequences were aligned together with 134 distinct SatDNA copies from eight *T*. *cruzi* stocks representing DTUs TcI-TcIII and TcV-TcVI, downloaded from GenBank (Table 1).

### Phylogenetic analysis

**Fig 1:**
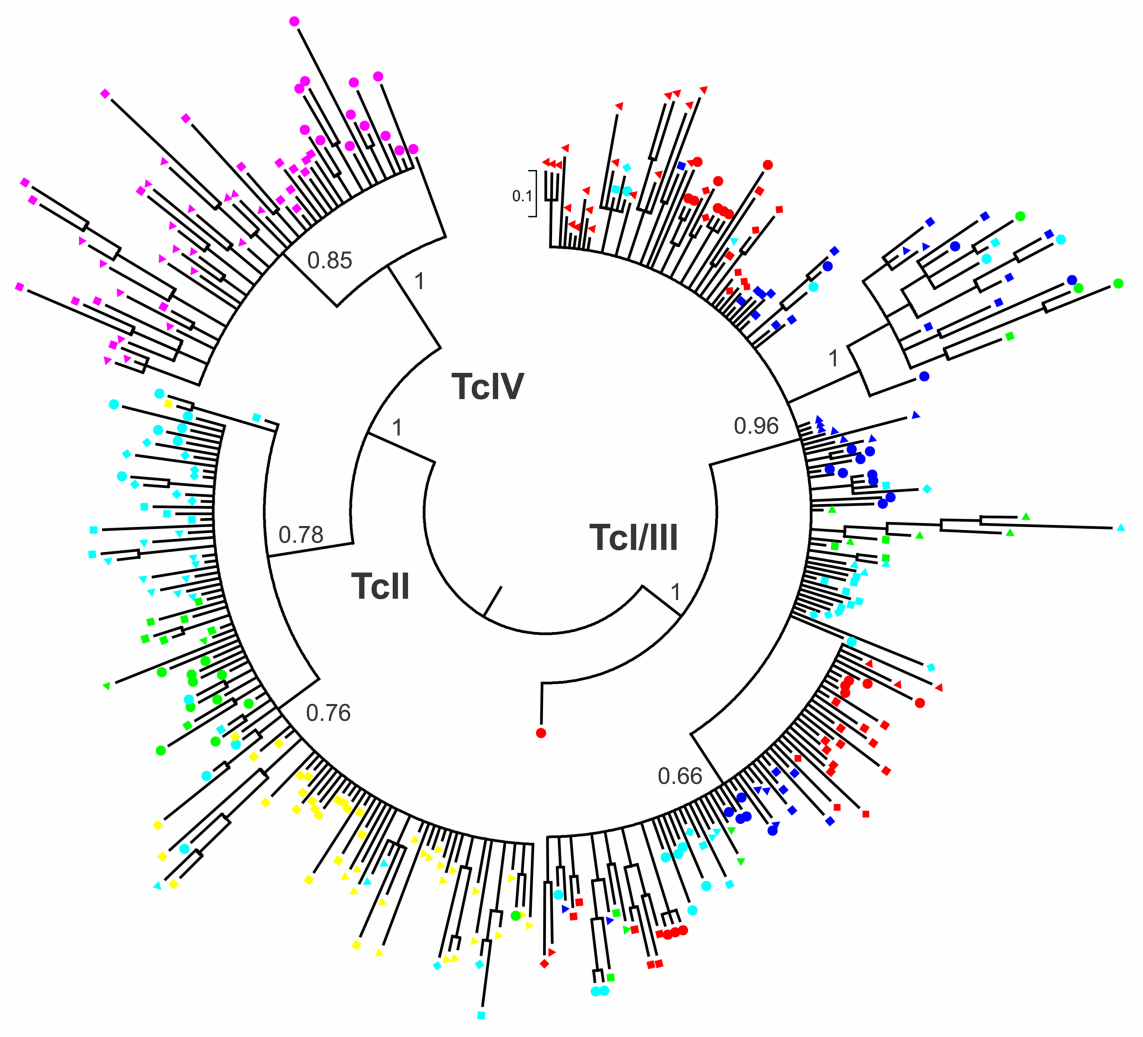
Bayesian phylogenetic tree of 335 satellite DNA sequences from 19 *T*. *cruzi* stocks representative of Discrete Typing Units TcI-TcVI. TcI [Sylvio X10 cl1 (▲), K-98 (■), Col4R (●), and Dm28c (♦) stocks], TcII [Y (▲) and JG (■) stocks], TcIII [3869 (▲), M6241 cl6 (■), and LL051-P24-Ro (●) stocks], TcIV [4167 (▲), Am64 (■), and Dog Theis (●) stocks], TcV [115 (▲), B147 (■), and NR cl3 (●) stocks], and TcVI [CL Brener (▲), RA (■), VD (●), and Tulahuen (♦) stocks]. Posterior probability values are shown at nodes for relevant groups.

Bayesian phylogenetic analysis showed that all 335 SatDNA sequences were grouped in three major clusters named TcI/III, TcII and TcIV (Fig 1), which corresponded to sequences from TcI and TcIII, TcII, and TcIV, respectively; whereas TcV and TcVI stocks had two sets of sequences distributed into TcI/III and TcII clusters. No monophyletic subgroup was found within TcIV cluster for sequences from TcIV_S_ (4167 and Am64) and TcIV_N_ (Dog Theis) stocks.

TcI/III was the cluster with more sequences (179), more than TcII (105) and TcIV (51) together, and also the one with the most complex topology; containing two main subgroups of 18 and 66 sequences. The first one was highly supported [posterior probability (PP) = 1] and comprised SatDNA sequences from all TcIII, two TcV (B147 and NR cl3) and two TcVI (RA and VD) stocks. The second one (PP = 0.66) was the most diverse and included sequences from all TcI, TcIII and TcVI, and two TcV (115 and B147) stocks.

Table 2 details the TcI/III and TcII clusters distribution of SatDNA sequences from TcV and TcVI hybrid *T*. *cruzi* stocks. As shown, while TcV stocks 115 and NR cl3 had the highest rates of SatDNA type TcI/III and TcII sequences, respectively, B147 showed a similar distribution of sequences into both clusters. In the case of TcVI stocks, RA and VD ranged between 60 and 40% of SatDNA type TcI/III and TcII sequences, respectively, Tulahuen showed the opposite distribution and CL Brener had twice more SatDNA type TcII than TcI/III sequences.

**Table 2:** Cluster distribution of satellite DNA sequences from TcV and TcVI stocks.

DTU	*T*. *cruzi* stock	No. of sequences	SatDNA type TcI/III [N (%)]	SatDNA type TcII [N (%)]
**TcV**	**115**	10	8 (80.0)	2 (20.0)
	**B147**	13	6 (46.2)	7 (53.8)
	**NR cl3**	14	3 (21.4)	11 (78.6)
**TcVI**	**CL Brener**	20	7 (35.0)	13 (65.0)
	**RA**	20	12 (60.0)	8 (40.0)
	**VD**	24	13 (54.2)	11 (45.8)
	**Tulahuen**	17	7 (41.2)	10 (58.8)

DTU: Discrete Typing Unit

N: number of sequences

Estimation of genetic distances [Mean of nucleotide substitutions per site (± Standard Error)] within clusters gave values of 5.6 (± 1.1), 4.0 (± 0.8) and 6.3 (± 1.1) % for TcI/III, TcII and TcIV clusters, respectively. On the other hand, the comparison between clusters gave higher distances between TcI/III and TcIV clusters [9.5 (± 2.9) %] than between TcI/III and TcII [7.3 (± 2.2) %], and TcII and TcIV clusters [6.9 (± 2.1) %].

When genetic distances were analyzed grouping sequences by *T*. *cruzi* DTUs, similar estimates were obtained within TcI [5.3 (± 1.1) %], TcII [4.0 (± 0.8) %], and TcIII [5.2 (± 1.1) %] compared to their corresponding clusters; whereas both TcV and TcVI had the highest value [8.6 (± 1.6) %]. As expected, a low genetic distance was found between sequences from TcI and TcIII stocks, and higher values were observed when they were compared with those from TcII stocks (Table 3). In general, TcV and TcVI sequences were very similar between them and closer to TcII than to TcI and TcIII sequences; whereas TcIV sequences were more distant to those from TcI and TcIII stocks than to TcII, TcV and TcVI sequences.

**Table 3:** Genetic distances of satellite DNA sequences between *T*. *cruzi* DTUs.

	Number of nucleotide substitutions per site [Mean (± SE)] (%)				
DTU/	TcI	TcII	TcIII	TcIV	TcV
**TcII**	**7.8** (± 2.3)				
**TcIII**	**0.7** (± 0.3)	**6.5** (± 2.1)			
**TcIV**	**9.7** (± 2.8)	**6.9** (± 2.0)	**9.7** (± 2.8)		
**TcV**	**2.8** (± 0.8)	**1.3** (± 0.4)	**2.1** (± 0.7)	**6.2** (± 1.8)	
**TcVI**	**2.2** (± 0.6)	**1.7** (± 0.5)	**1.6** (± 0.5)	**6.2** (± 1.8)	**0.0** (± 0.1)

DTU: Discrete Typing Unit

SE: Standard Error

### Signature pattern analysis

The signature patterns identified for the three clusters of SatDNA sequences are shown in Table 4.

**Table 4:** Signature patterns of *T*. *cruzi* satellite DNA sequences.

SatDNA type	Polymorphic sites^a^ and nucleotide frequencies^b^														
	7	16	33	58	73	85	87	88	100	116	121	125	127	128	129
**TcI/III**	**T** (0.77)	**A** (0.91)	**G** (0.97)	**C** (1)	**T** (0.98)	**T** (0.98)	**G** (0.94)	**A** (1)	**C** (0.89)	**T** (1)	**T** (0.97)	**A** (0.96)	**G** (0.98)	**T** (1)	**A** (0.88)
	**C** (0.22)	**C** (0.06)	**T** (0.03)	---	**C** (0.02)	**G** (0.01)	**C** (0.05)	---	**T** (0.11)	---	**G** (0.03)	**C** (0.03)	**A** (0.02)	---	**C** (0.12)
**TcII**	**T** (1)	**C** (0.72)	**T** (1)	**T** (0.98)	**C** (0.73)	**T** (1)	**C** (0.98)	**G** (0.84)	**T** (1)	**T** (0.97)	**G** (0.99)	**A** (0.78)	**A** (1)	**T** (1)	**C** (1)
	---	**A** (0.26)	---	---	**T** (0.25)	---	---	**A** (0.16)	---	**A** (0.03)	**T** (0.01)	**C** (0.22)	---	---	---
**TcIV**	**C** (0.86)	**G** (0.98)	**G** (1)	**T** (0.88)	**C** (0.63)	**G** (0.84)	**G** (0.84)	**A** (1)	**T** (1)	**A** (1)	**G** (0.88)	**C** (1)	**A** (0.98)	**C** (0.84)	**G** (0.92)
	**T** (0.14)	**A** (0.02)	---	**C** (0.12)	**T** (0.37)	**T** (0.08)	**C** (0.16)	---	---	---	---	---	**G** (0.02)	**T** (0.16)	**C** (0.02)

^a^Only relevant nucleotides are shown at each site

^b^Nucleotide frequencies are indicated in parentheses

Fifteen polymorphic sites associated to one or two types of SatDNA sequence were found. All together, they defined a specific signature pattern for each type of SatDNA sequence (TcI/III, TcII and TcIV). Only for two sites (16 and 129) the predominant nucleotide was able to resolve among the three types of SatDNA sequences, but even in these sites it was possible to find sequences from one cluster with a less frequent nucleotide that was predominant in sequences from another cluster.

To validate these findings, the consensus sequence of each *T*. *cruzi* stock was obtained and the result of SatDNA type classification was compared with its corresponding DTU (Table 5). For each consensus sequence, the polymorphic sites associated to a particular SatDNA type were counted and the sequences classified on the basis of the SatDNA type that reached the highest score. For homozygous lineages (TcI-TcIV), SatDNA type classification completely matched with the reported DTU of each *T*. *cruzi* stock; although, as expected, for TcI and TcIII stocks it was not possible to resolve between both DTUs and, in consequence, they were classified as SatDNA type TcI/III. Similarly, *T*. *cruzi* stocks of heterozygous lineages (TcV and TcVI), harboring both types of SatDNA sequences TcI/III and TcII, could not be assigned to a specific DTU and were classified as SatDNA type Hybrid.

**Table 5:** Signature patterns and classification of satellite DNA consensus sequences from the 19 *T*. *cruzi* stocks analyzed in this work.

		Polymorphic sites^a^															Sites associated to^b^			SatDNA
*T*. *cruzi* stock	DTU	7	16	33	58	73	85	87	88	100	116	121	125	127	128	129	TcI/III	TcII	TcIV	type
**Sylvio X10 cl1**	**TcI**	T/C	A	G	C	T	T	G	A	C	T	T	A	G	T	A	**15**	5	4	**TcI/III**
**K-98**		T/C	A	G	C	T	T	G	A	C	T	T	A	G	T	A	**15**	5	4	**TcI/III**
**Col4R**		T/C	A	G	C	T	T	G	A	C	T	T	A/C	G/A	T	A/C	**15**	7	6	**TcI/III**
**Dm28c**		T	A	G	C	T	T	G	A	C	T	T	A	G	T	A	**15**	5	3	**TcI/III**
**Y**	**TcII**	T	C/A	T	T	C/T	T	C	G	T	T	G	A/C	A	T	C	7	**15**	6	**TcII**
**JG**		T	C/A	T	T	C/T	T	C	G/A	T	T	G	A	A	T	C	8	**15**	6	**TcII**
**3869**	**TcIII**	T	A/C	G	C	T	T	G	A	C	T	T	A	G	T	A	**15**	8	4	**TcI/III**
**M6241 cl6**		T	A/C	G	C	T	T	G	A	C/T	T	T	A	G	T	A/C	**15**	6	3	**TcI/III**
**LL051-P24-Ro**		T/C	A	G	C	T	T	G	A	C	T	T	A	G	T	A	**15**	5	4	**TcI/III**
**4167**	**TcIV**	C	G	G	T	C/T	G	G	A	T	A	G	C	A	C/T	G	5	6	**15**	**TcIV**
**Am64**		C	G	G	T/C	C/T	G	G/C	A	T	A	G	C	A	C	G	5	6	**15**	**TcIV**
**Dog Theis**		C	G	G	T	C/T	G	G	A	T	A	G	C	A	C	G	4	5	**15**	**TcIV**
**115**	**TcV**	T	A	G/T	C/T	T/C	T	G/C	A/G	C/T	T	T/G	A	G/A	T	A/C	**13**	**15**	8	**Hybrid**
**B147**		T	A/C	T/G	T/C	T/C	T	C/G	A/G	T/C	T	G/T	A	A/G	T	C/A	**15**	**14**	8	**Hybrid**
**NR cl3**		T	C/A	T/G	T/C	C/T	T	C/G	G/A	T	T	G/T	A	A/G	T	C	**15**	**15**	8	**Hybrid**
**CL Brener**	**TcVI**	T	A/C	T/G	T/C	T/C	T	C/G	A/G	T/C	T	G/T	A/C	A/G	T	C/A	**15**	**15**	8	**Hybrid**
**RA**		T	A/C	G/T	C/T	T/C	T	G/C	A/G	C/T	T	T/G	A	G/A	T	A/C	**15**	**15**	8	**Hybrid**
**VD**		T/C	A/C	G/T	C/T	T/C	T	C/G	A/G	T/C	T	T/G	A/C	G/A	T	C/A	**15**	**15**	10	**Hybrid**
**Tulahuen**		T	C/A	T/G	T/C	T/C	T	C/G	G/A	T/C	T	G/T	A	A/G	T	C/A	**15**	**15**	9	**Hybrid**

DTU: Discrete Typing Unit

^a^The presence of two nucleotides indicates the most/least frequent ones

^b^Out of a total of 15 polymorphic sites

Hybrid: sequences from TcV or TcVI stocks with signature patterns of both satellite DNA types TcI/III and TcII

### Primers design and SatDNA qPCR analysis

**Fig 2:**
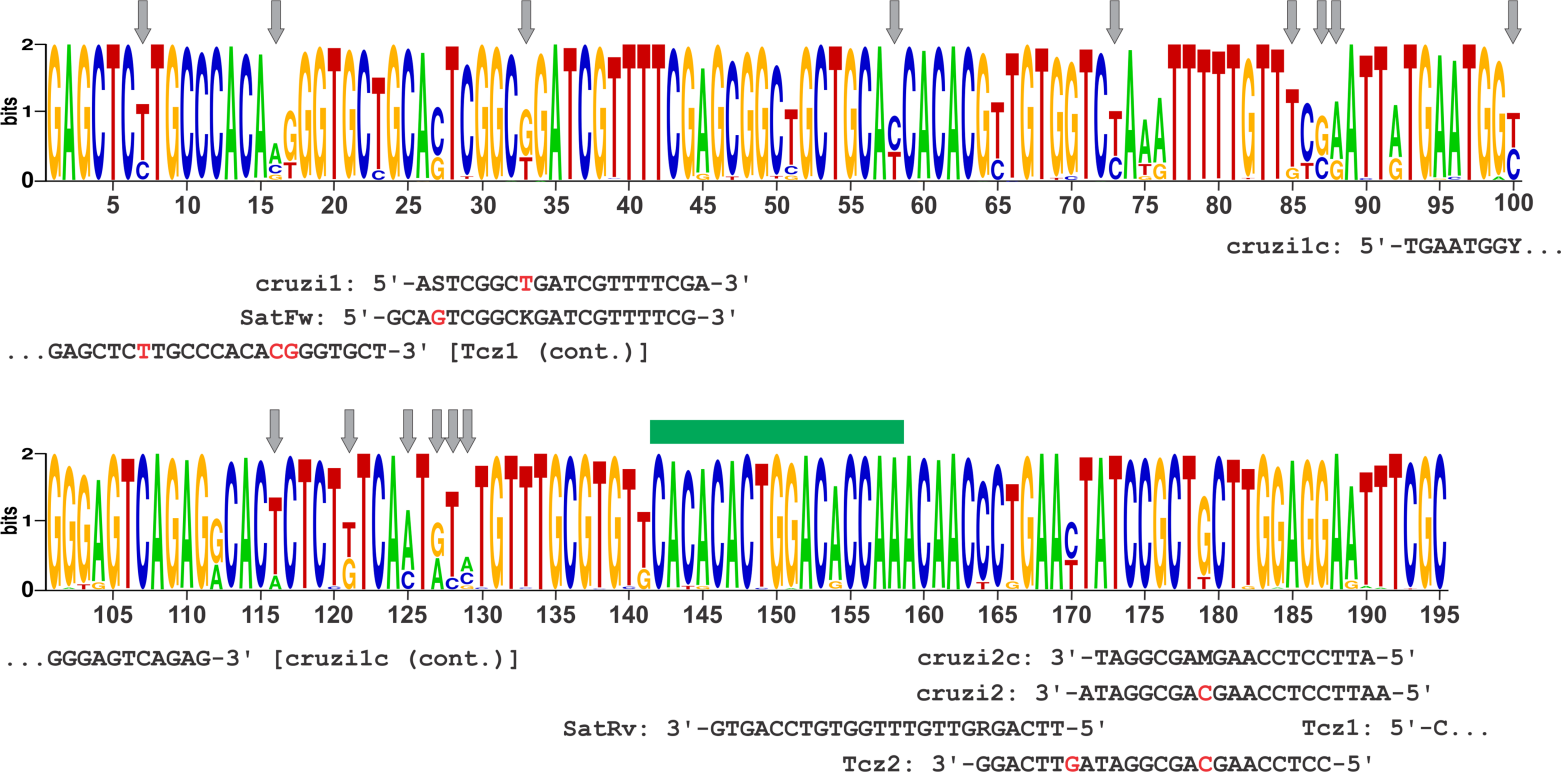
Graphic representation of the consensus sequence of 335 satellite DNA sequences from 19 *T*. *cruzi* stocks representative of Discrete Typing Units TcI-TcVI. The target sequences of Tcz1/Tcz2, SatFw/SatRv, cruzi1/cruzi2 and the novel cruzi1c/cruzi2c primers are shown. Arrows point to the 15 polymorphic sites associated to satellite DNA signature patterns. Horizontal bar indicates the binding site of cruzi3 TaqMan probe.

Fig 2 shows the graphic representation of the consensus sequence of the 335 SatDNA sequences analyzed in this work. Based on the conserved regions and polymorphic sites associated to SatDNA types, cruzi1c and cruzi2c primers were designed to amplify a segment of 98 bp, aimed to be used in a SatDNA qPCR assay, with cruzi3 TaqMan probe, for molecular diagnostic purposes.

The novel cruzi1c/cruzi3/cruzi2c SatDNA qPCR assay was compared with the validated cruzi1/cruzi3/cruzi2 qPCR method, against a set of dilutions of genomic DNA from 12 *T*. *cruzi* stocks representing DTUs TcI-TcVI (Table 6). As shown, the new qPCR assay was more sensitive than the previous method for all *T*. *cruzi* DTUs, except for TcII stocks, for which both qPCRs gave similar sensitivity. In particular, the most remarkable differences between the results of both qPCR assays were found for Sylvio X10 cl1 (TcI), 4167 (TcIV), Am64 (TcIV), and MN cl2 (TcV) stocks.

**Table 6:** Comparative analysis of *T*. *cruzi* satellite DNA qPCR sensitivity using cruzi1/cruzi2 and the novel cruzi1c/cruzi2c primers.

		qPCR cruzi1/cruzi2—*T*. *cruzi* DNA (fg/μL)					qPCR cruzi1c/cruzi2c—*T*. *cruzi* DNA (fg/μL)				
DTU	*T*. *cruzi* stock	0.0625	0.125	0.25	0.5	1.0	0.0625	0.125	0.25	0.5	1.0
**TcI**	**Sylvio X10 cl1**	ND	ND	ND	ND	ND	ND	ND	Pos	Pos	Pos
** **	**K-98**	ND	ND	ND	Pos	Pos	ND	ND	Pos	Pos	Pos
**TcII**	**Y**	ND	Pos	Pos	Pos	Pos	ND	Pos	Pos	Pos	Pos
** **	**JG**	Pos	Pos	Pos	Pos	Pos	Pos	Pos	Pos	Pos	Pos
**TcIII**	**M5631 cl5**	ND	Pos	Pos	Pos	Pos	Pos	Pos	Pos	Pos	Pos
** **	**LL051-P24-Ro**	ND	ND	Pos	Pos	Pos	ND	Pos	Pos	Pos	Pos
**TcIV**	**4167**	ND	ND	ND	ND	Pos	Pos	Pos	Pos	Pos	Pos
** **	**Am64**	ND	ND	ND	ND	Pos	ND	Pos	Pos	Pos	Pos
**TcV**	**MN cl2**	ND	ND	ND	ND	Pos	Pos	Pos	Pos	Pos	Pos
** **	**PAH179**	ND	Pos	Pos	Pos	Pos	Pos	Pos	Pos	Pos	Pos
**TcVI**	**CL Brener**	ND	ND	Pos	Pos	Pos	Pos	Pos	Pos	Pos	Pos
	**RA**	ND	Pos	Pos	Pos	Pos	Pos	Pos	Pos	Pos	Pos

ND: non-detectable

Pos: positive

## Discussion

### Phylogenetic inference

Two major evolutionary models have been proposed to explain the origin of *T*. *cruzi* hybrid lineages, but while it is widely accepted that TcV and TcVI are the result of genetic exchange between TcII and TcIII strains, the origin of TcIII and TcIV is still a matter of debate. Thereby, the main difference between the Two Hybridization [12] and the Three Ancestor [13] models is whether or not TcIII and TcIV were originated from a hybridization event between TcI and TcII strains, respectively. Accordingly, the acquisition of TcI alleles via TcIII by TcV and TcVI hybrid lineages is supported by some authors [8,12,25], whereas others have found no evidence of it [13,14,38]. Likewise, previous analysis of SatDNA sequences have also shown contradictory findings concerning to the existence of SatDNA type TcII sequences in TcIII isolates [23,24]; whereas no TcIV sequence has been analyzed yet. In the present work, we have gone deeper into this matter analyzing 335 distinct SatDNA sequences from 19 *T*. *cruzi* stocks representing DTUs TcI-TcVI, including TcIV_S_ and TcIV_N_ isolates (Table 1).

All TcI and TcIII sequences were grouped in the same cluster, whereas TcV and TcVI sequences were distributed into TcI/III and TcII clusters (Fig 1), as previously described [24]. The fact that none TcIII sequence clustered with TcII sequences and that all TcIV sequences were grouped in a unique and independent cluster support the theory that TcIII and TcIV are not the result of a hybridization event between TcI and TcII, in concordance with the Three Ancestor model [13] and other authors [14,38]. The highly supported monophyletic subgroup including TcIII but not TcI sequences found in TcI/III cluster may reflect a remnant of an ancestral hybridization event during the origin of TcIII; however, no evidence was found that it could involve TcII strains, as previously proposed [24]. Although we cannot deny this possibility, it seems unlikely that TcII SatDNA fingerprints have been smudged in current TcIII strains, whereas the remnant of the ancestral hybridization event observed in TcIII have also been found in TcV and TcVI stocks.

The closeness between TcI and TcIII sequences found by phylogenetic inference and genetic distance estimation (Table 3) is in concordance with previous analysis of nuclear genome data indicating that both DTUs share a common ancestor [14] or the participation of TcI in an ancestral hybridization event yielding TcIII [24]. On the other hand, the fact that TcIV does not share any SatDNA sequence with TcI and TcIII, and vice versa, and the highest genetic distances found between them, contrast with the theory that these three DTUs emerged from the same ancestor [14]. It is worth noting that TcIV sequences were not split up into TcIV_S_ and TcIV_N_ subgroups, as it has been described for other genetic markers [9,14]; although this DTU was found as the homozygous lineage with the highest intra-DTU diversity. Concerning to TcI, despite its well-known genetic diversity [39,40] and the fact that four isolates from different geographic regions were analyzed, this lineage showed an intermediate intra-DTU distance. Finally, the lowest genetic diversity of TcII is in concordance with previous analysis of other nuclear sequences [9], but it could also be due to the fact that only two TcII isolates were included in this work and both came from the same geographic region.

The presence of TcII and TcIII SatDNA fingerprints in TcV and TcVI stocks, supports the accepted theory that both DTUs are the result of genetic exchange between TcII and TcIII strains [9,12,13]. The heterozygosity and the common parental ancestors of TcV and TcVI explain the fact that both lineages had the highest intra-DTU diversity and showed the lowest genetic distance between *T*. *cruzi* DTUs (Table 3). The high diversity of SatDNA sequences within TcV and TcVI contrasts with the homogeneity found in both DTUs analyzing other nuclear markers [9], possibly due to that SatDNA belongs to the fast-evolving portion of eukaryotic genomes [41]. The different rates of SatDNA type TcI/III and TcII sequences among TcV and TcVI stocks (Table 2), may reflect: i) independent clonal evolution since both DTUs were originated, ii) several independent hybridization events between different TcII and TcIII strains that led to TcV and TcVI strains with distinct SatDNA content, iii) genetic exchange between hybrid progeny and parental lineages, or iv) a combination of these and other possible scenarios.

### Typing and diagnostic applications

The remarkable genetic diversity as well as the different geographic distribution and transmission cycles of *T*. *cruzi* DTUs make their identification a matter of great interest for ecological, epidemiological and clinical studies [2,3]. Several strategies have been proposed to genotype *T*. *cruzi* isolates but, due to sensitivity constraints, most of these methods have been applied only to cultured stocks and biological or clinical samples with high parasitic loads [26,42–47]. The low sensitivity of these strategies resides in the single or low copy number of their target sequences, therefore the significant impact that the use of molecular targets with high copy number like SatDNA sequence may have on clinical and epidemiological genotyping studies.

No consensus motifs of SatDNA sequence have been found for any *T*. *cruzi* DTU [24]. Following a different approach, we have identified specific SatDNA TcI/III, TcII and TcIV signature patterns (Table 4). Although further validation will be necessary to implement SatDNA typing, the perfect coincidence between SatDNA classification and the reported DTU for 19 *T*. *cruzi* stocks (Table 5) supports its application in genotyping studies; principally when the usual methods fail. The major limitation of this approach is that it cannot distinguish between the presence of hybrid lineages TcV and TcVI, and the existence of mixed infections with TcI or TcIII and TcII strains. However, in these cases the epidemiological characteristics of the region from where the sample is taken may help to fill this gap. This typing strategy could be particularly useful for chronic Chagas disease patients with low parasitic loads, whose samples are usually very difficult to genotype [26,48]. Indeed, a first version of this approach allowed the characterization of samples from chronic patients that gave non-detectable results using traditional genotyping methods [49].

Based on the polymorphism of minicircle hypervariable regions, a highly repetitive sequence from kinetoplastid DNA (kDNA), a minicircle lineage-specific PCR assay has been developed to detect the presence of single or mixed infections of TcI, TcII, TcV and TcVI in clinical samples [50,51]. Considering that the analysis of SatDNA or kDNA sequences does not allow the identification of all *T*. *cruzi* DTUs, as for most molecular markers, a combined strategy using both repetitive sequences might help to fully resolve the genotyping of clinical samples with low parasitic loads.

Due to its high copy number, SatDNA sequence has been one of the most used targets for molecular diagnostics of *T*. *cruzi* infection [16–20]. However, the most used primers for conventional PCR (Tcz1/Tcz2) [52], Sybr Green real-time PCR (SatFw/SatRv) [18], and TaqMan real-time PCR (cruzi1/cruzi2) [17] approaches based on SatDNA amplification were designed long time ago when few sequences from TcI, TcII and TcVI, and almost none from TcIII, TcIV and TcV isolates were available. Therefore, we were interested in revising the suitability of these primers and, in case of being necessary, designing new ones. Except for SatRv, all the target sequences of these primers include polymorphic sites that were not considered in their design (Fig 2). In particular, the target sequences of Tcz1 and cruzi1 include polymorphic sites associated to specific SatDNA types and, in consequence, could be leading to misdiagnose infections with some TcI, TcIII and TcIV strains; principally in patients with low parasitic loads.

During the design of cruzi1c and cruzi2c primers, the amplicon size was reduced to improve TaqMan qPCR efficiency, as recommended [53]. Both considerations, avoiding polymorphic sites and reducing amplicon size, led to an improved sensitivity of SatDNA qPCR assay (Table 6). It is worth noting the higher sensitivity of the new qPCR assay against TcIV and some TcI and TcV stocks, compared to the validated qPCR method [20]. TcIV, traditionally associated with the sylvatic cycle and occasional oral outbreaks due to food contamination [2,3], was recently found as the second more frequent DTU in Bolivian chronic Chagas disease patients living in Madrid, Spain [54]; indicating that the incidence of this DTU in clinical cases may have been underestimated. The lowest sensitivity of both qPCR methods against TcI stocks was probably due to the lowest copy number of SatDNA sequence observed in strains from this DTU [55]. Analytical and clinical validation studies will be necessary before using the new qPCR assay for the molecular diagnostics of *T*. *cruzi* infection.

Summarizing, our findings support the theory that TcIII is not the result of a hybridization event between TcI and TcII, and that TcIV had an independent origin from the other DTUs, contributing to clarifying the evolutionary history of *T*. *cruzi* lineages. Moreover, this work opens the possibility of typing samples from Chagas disease patients with low parasitic loads and improving molecular diagnostic methods of *T*. *cruzi* infection based on SatDNA sequence amplification.

## Supporting information

S1 Dataset *T*. *cruzi* satellite DNA sequences analyzed in this work. (RAR)
